# The peroxidase PRDX1 inhibits the activated phenotype in mammary fibroblasts through regulating c-Jun N-terminal kinases

**DOI:** 10.1186/s12885-019-6031-4

**Published:** 2019-08-16

**Authors:** Agnieszka Jezierska-Drutel, Shireen Attaran, Barbara L. Hopkins, John J. Skoko, Steven A. Rosenzweig, Carola A. Neumann

**Affiliations:** 1 Department of Pharmacology & Chemical Biology, University of Pittsburgh, Pittsburgh, PA 15213 USA; 2 Department of Cell and Molecular Pharmacology & Experimental Therapeutics, Medical University of South Carolina, Charleston, SC 29425 USA; 3 Department of Human Genetics, University of Pittsburgh, Pittsburgh, PA 15213 USA; 4 Women’s Cancer Research Center, UPMC Hillman Cancer Center, Pittsburgh, PA 15213 USA

**Keywords:** peroxiredoxin1, Mammary fibroblasts, Cancer-associated fibroblasts, Breast cancer, Hydrogen peroxide, JNK, Breast tumorigenesis

## Abstract

**Background:**

Reactive oxygen species (ROS), including hydrogen peroxide, drive differentiation of normal fibroblasts into activated fibroblasts, which can generate high amounts of hydrogen peroxide themselves, thereby increasing oxidative stress in the microenvironment. This way, activated fibroblasts can transition into cancer-associated fibroblasts (CAFs).

**Methods:**

Mammary fibroblasts from either female 8 weeks old PRDX1 knockout and wildtype mice or Balb/c mice were studied for characteristic protein expression using immunofluorescence and immunoblotting. Cancer-associated fibroblasts was examined by transwell migration and invasion assays. The binding of PRDX1 to JNK1 was assessed by co-immuneprecipitation and JNK regulation of CAF phenotypes was examined using the JNK inhibitor SP600125. Extracellular hydrogen peroxide levels were measured by chemiluminescence via the reaction between hypochlorite and luminol. Statistical analyses were done using Students t-test.

**Results:**

We show here PRDX1 activity as an essential switch in regulating the activated phenotype as loss of PRDX1 results in the development of a CAF-like phenotype in mammary fibroblasts. We also show that PRDX1 regulates JNK kinase signaling thereby inhibiting CAF-like markers and CAF invasion. Inhibition of JNK activity reduced these behaviors.

**Conclusions:**

These data suggest that PRDX1 repressed the activated phenotype of fibroblasts in part through JNK inhibition which may present a novel therapeutic option for CAF-enriched cancers such as breast cancer.

**Electronic supplementary material:**

The online version of this article (10.1186/s12885-019-6031-4) contains supplementary material, which is available to authorized users.

## Background

Breast cancer is the most commonly occurring cancer in US women, as 1 in 8 will develop breast cancer in her lifetime. While hormonal therapies have proven effective, many cases are not responsive, develop drug resistance, or relapse at a later point, focusing a need for better prevention of later stage disease and metastasis. Breast cancer progression and metastasis include not only cell-autonomous properties of cancer epithelial cells but also the influence of the neighboring tumor stromal cells. In breast cancer, almost 80% of mammary fibroblasts (MFs) acquire a cancer-associated fibroblast (CAF)-like “activated phenotype”. This includes enhanced expression of growth factors, cytokines, enzymes degrading the extracellular matrix such as matrix metalloproteinases (MMPs), fibroblast activated protein (FAP) and mesenchymal markers such as alpha smooth muscle actin (α-SMA) that render target cells highly mobile. Although it is known that the CAF-activated phenotype is associated with elevated levels of reactive oxygen species (ROS) that are also linked with tumor spreading, exact mechanisms on how ROS promote CAFs are unknown [1–4].

Fibroblasts are the most abundant cell type in connective tissue. There, they form the structural network by secreting extracellular matrix components. During tissue remodeling, quiescent fibroblasts undergo activation and transition into activated fibroblasts, also termed myofibroblasts, which during wound healing and fibrosis gain contractile fibers by expressing α-SMA and form cell-cell contacts through gap junctions. Once the wound is closed, activated fibroblasts or myofibrobalsts undergo cell death in the form of apoptosis. In processes of fibrosis or cancer, however, activated fibroblasts fail to undergo apoptosis resulting in excess of ECM (desmoplasia) that promotes fibrosis and tumorigenesis. Oxidation processes induced by oxidants such as hydrogen peroxide (H_2_O_2_) have been identified as an essential contributor in the progression of normal fibroblasts into activated fibroblasts and importantly, promote the transition into CAFs [2–8]. Mechanistic details on how oxidants promote the CAF phenotype are still unclear though, therefore, as the mammary gland is a stroma rich organ, elucidation of signaling proteins regulated by H_2_O_2_ is essential to combat CAF function in breast cancer.

Peroxiredoxin (PRDX) family members (typical 2-Cys: PRDX1–4, atypical 2-Cys: PRDX5 and 1-Cys: PRDX6) are antioxidant enzymes that reduce peroxides via catalytic cysteine oxidation to sulfenic acid. 2-Cys PRDXs are important redox sensors in signaling through two unique mechanisms: a) they possess a highly reactive catalytic cysteine that converts to a protein sulfenic acid moiety resulting in a disulfide bond with a resolving cysteine, which can be reduced via thioredoxin to reset catalytic function [9]; and b) during recycling where the catalytic cysteine of PRDX can be further oxidized, which promotes in turn formation of PRDX decamers that display chaperone functionality, but lack peroxidase activity [10]. These features enable PRDX1 to sense and react to changes in redox signaling by controlling protein-binding partners. Research suggests that PRDXs play roles in cancer prevention and progression, however, their functions may be isoform and tissue-specific [11].

The family of c-Jun NH_2_-terminal kinases comprises three family members (JNK1–3), where JNK1 and JNK2 are ubiquitously expressed, and JNK3 is predominantly found in the brain [12]. Like other MAP kinases, JNKs are activated by reactive oxygen species and signal to a complex protein network inducing apoptosis, as well as cell survival, migration and invasion depending on cellular context [13]. PRDX1 associates with the Glutathione *S*-Transferase Pi/JNK complex, thereby suppressing JNK activation during ionizing radiation [14]. JNK signaling regulates apoptosis, proliferation and as other recent studies report cell motility. JNK activation by various growth factors induces cell migration in many cell types, which is repressed by the JNK-inhibitor SP600125 [reviewed in [15]]. JNKs phosphorylate and regulate the transcription factor and proto-oncogene c-jun that when deleted, reduces migration and invasiveness of different cell types [16, 17]. Supporting a role for JNK in stromal tissues, JNK1 has been shown to promote lung fibrosis in vivo, and to control ROS production through NADPH oxidase 4 (NOX4) thus inducing myofibroblast differentiation and upregulation of alpha-smooth muscle actin (α-SMA) in human breast stromal cells [18, 19]. Hyperactive JNK1 signaling is also associated with high breast density which is characterized by increases in glandular and fibrous mammary tissue [20].

A recent study placed JNK downstream of the PTEN tumor suppressor in mammary fibroblasts. Accordingly, targeted loss of PTEN in mammary fibroblasts promoted phenotypes of CAFs and breast cancer development in mice correlating with increased activation of JNK [21]. Both, PTEN and JNK are PRDX1-interacting proteins, and while PRDX1 promotes PTEN activity, it inhibits JNK activity under conditions of ionizing radiation [14, 21]. Therefore, as loss of PRDX1 peroxidase activity has been implicated in fibroblast wound closure [22], we hypothesized that PRDX1 might play a role in mammary fibroblast signaling through PTEN. Here, we show that the lack of PRDX1 promotes characteristics found in cancer-associated fibroblasts (CAFs). Also, our data suggest PRDX1 inhibits JNK, in turn decreasing the expression of α-SMA and invasive phenotypes of mammary CAFs. While these novel data imply that targeting JNK may be a strategy to inhibit tumor desmoplasia, they also suggest that targeting PRDX1 in cancer could promote unwanted tumor desmoplasia.

## Methods

### Cell culture

All MF cell lines were cultured in DMEM (Mediatech) supplied with 5% FBS (HyClone), 100 units/ml penicillin, 100 mg/ml streptomycin (Mediatech) and 2 mM l-glutamine (Mediatech) (complete DMEM) at 37 °C in a 5% CO_2_ and 5% oxygen. 293 T (HEK 293 T) cells were obtained from ATCC and grown in DMEM (Mediatech) supplied with 10% FBS (HyClone), 100 units/ml penicillin, 100 mg/ml streptomycin (Mediatech) and 2 mM l-glutamine (Mediatech) (complete DMEM) in a 37 °C incubator supplied with 5% CO_2_ in atmospheric oxygen.

### Plasmids

pcDNA3-FLAG-JNK1a1 has previously been described [23] and was purchased from Addgene. Human shPRDX1 expression constructs were recently described [24], and the murine shPRDX1 expression constructs were either designed using GPP Web Portal of the Broad Institute (http://portals.broadinstitute.org/gpp/public/) or purchased from Sigma Aldrich.

### Isolation of murine mammary fibroblasts

*Prdx1*^*−/−*^ and *Prdx1*^*+/+*^ control mice of the same genetic background (C57BL6/N × 129/sV) were produced as previously described [25]. Female BALB/c mice were purchased from Jackson Laboratory. Animals used for this study were approved by and conducted according to the guidelines of the University of Pittsburgh IACUC. Mammary fibroblasts (MFs) were isolated from 8 to12-week old virgin female mice of each genotype (*Prdx1*^*−/−*^ and *Prdx1*^*+/+*^ mice) or BALB/c wild-type. Briefly, mice were sacrificed using a CO_2_ overdose, and the mammary glands quickly removed, washed twice in wash solution (46 mL Dulbecco’s phosphate buffered saline (DPBS) (Sigma), 2.5 mL FBS (Gibco), 100 units/ml penicillin, 100 mg/ml streptomycin (Mediatech) and 400 μL Fungizone (Thermofisher), and finely minced. Tissues were then disaggregated by repeated aspiration using a 10 ml syringe (no needle). Tissues were then centrifuged and digested at 37 °C for 2 h in DMEM containing 10% FBS, 100 units/ml penicillin, 100 mg/ml streptomycin (Mediatech), 3500 units/ml collagenase followed by a 10 min trypsin digestion step that was neutralized with FBS. Cells were then washed twice in PBS and plated in complete DMEM with 5% FBS. After 2 h, non-adherent cells were removed, and the remaining fibroblasts were cultured for several weeks at 37 °C in a 5% CO_2_ and 5% oxygen until spontaneously immortalized.

### Immunofluorescence

MEFs or MFs seeded on glass coverslips were fixed for 15 min in 3.7% paraformaldehyde, rinsed twice in cold PBS pH 7.4 for 10 min and permeabilized in blocking solution (PBS with 5% BSA and 0.3% Triton™ X-100) for 30 min. Coverslips were then washed twice in chilled PBS pH 7.4 for 10 min and specific primary antibodies (anti-collagen-1 (Calbiochem), α-smooth muscle actin - Cy5 (Sigma-Aldrich), vimentin (Cell Signaling) were diluted 1:250 in antibody dilution buffer: (PBS with 5% BSA and 0.3% Triton™ X-100) were applied overnight at 4 °C. Cells were washed twice in cold PBS pH 7.4 for 10 min, and fluorochrome-conjugated secondary antibody (mouse or rabbit) Alexa Fluor® (Molecular Probes, Life Technologies) diluted 1:2000 in antibody dilution buffer were applied for 2 h at RT in the dark. To visualize DNA, after two 10-min washes, cells were stained with Hoechst 33258 (Molecular Probes, Life Technologies) for 15 min at RT in the dark. The slides were again rinsed in PBS and then the coverslips were mounted on microscope slides using Prolong® Gold Anti-Fade Reagent (Molecular Probes, Life Technologies). Slides were imaged on a BD Biosciences CARV II Confocal Imager.

### Immune precipitations

HEK 293 T cells (5 × 10^5^) were transiently transfected with 2 μg pcDNA3-FLAG-JNK1a1 or control plasmid, using the Fugene 6 system for 48 h. Cells were serum starved for 30 min, then treated with 0, 25 or 250 μM H_2_O_2_ for 30 min. Before lysis, cells were washed one time with PBS containing 20 mM NEM (N-ethylmaleimide) to avoid oxidation of free thiols. Samples were lysed using a Tris lysis buffer (50 mM Tris; 2% Triton X-100; 0.5 mM EDTA; 0.5 mM EGTA; 150 mM NaCl; 10% glycerol; 50 mM NaF; 1 mM NaVO_4_; 40 mM β-glycerophosphate), supplemented with 30 μg/ml catalase from bovine liver (Sigma), and proteinase inhibitors. Protein concentrations were quantified using the Pierce BCA Protein Assay kit, according to the manufacturer’s instructions (Thermo). 1 mg of cell lysate was incubated with 20 μL of acid treated Anti-FLAG M2 Affinity Gel (Sigma) and 400 μL lysis buffer, at 25 °C for 3 h, with rotation. Immunoadsorbed samples were collected and washed four times with lysis buffer, and once with TBS. Beads were boiled in Laemmli sample buffer (BioRad) in the presence or absence of β-mercaptoethanol (Sigma) for 10 min. 20 μg of whole cell lysate input was prepared in Laemmli sample buffer as above for 5 min.

### Immunoblotting

Prepared, immunoprecipitated (IP) samples and corresponding whole cell lysates were resolved by SDS-PAGE and transferred to a nitrocellulose membrane according to the manufacturer’s (BioRad) instructions. Membranes were blocked with 5% BSA in TBS for 30 min, and incubated with primary antibodies against JNK1/2 (1:1000)(Invitrogen), P-SAPK/JNK (1:1000) (Cell Signaling), P-c-jun (1:1000) (Cell Signaling), P-ATF2(1:1000) (Cell Signaling), PRDX1 (1:4000)(Abcam), PRDX-SO3 (1:1000)(Abcam), and actin (1:1000) (Oncogene), overnight at 4 **°**C. Membranes were washed four times for 5 min each in TBST (0.05% Tween-20) and visualized by IR or chemiluminescent detection. For IR processing, membranes were incubated with a 1:15000 dilution of anti-goat, anti-rabbit, or anti-mouse IRDye (LI-COR), for 30 min at 25 °C. Blots were washed three times with TBST, once with TBS, and imaged on an Odyssey (LI-COR) imager. Membranes processed by chemiluminescence were incubated in a 1:10000 dilution of HRP-conjugated anti-mouse or anti-rabbit antibodies for 1 h at 25 °C. Blots were washed four times with TBST for 5 min and exposed to ECL for 1 min.

### Cell treatment with H_2_O_2_ and JNK inhibitors

Before the individual experiments, cells were starved for 48 h in 0.25% FBS DMEM and pretreated for 1–2 h with 25 μM JNK inhibitor, SP600125 (Tocris Bioscience), 2 μM JNK-IN-8 (a kind gift from Dr. Nathanial Grey, DFCI, Boston) or 10 μM API-1 (Tocris Bioscience). Hydrogen peroxide was added for 10 min or 48 h depending on the experiment.

### Migration assay

MFs were starved in 0.25% FBS DMEM for 24 h at 37 °C, 5% CO_2_. Cells were trypsinized, centrifuged for 5 min at 1500 g and suspended in 0.25% FBS DMEM. Cells were counted, and 3 × 10^4^ fibroblasts in 0.25% FBS DMEM were seeded in the self-standing Millicell Culture Plate Inserts (Millipore). 2 ml of 10% FBS DMEM was added to the plate. The migration assay was allowed to proceed for 24 h at 37 °C, 5% CO_2_. A damp cotton swab was used to wipe the non-migrated cells from the top of the transwell membrane. Cells were fixed with 3.7% paraformaldehyde for 15 min, washed twice with PBS and stained with 0.1% crystal violet. The number of migrating MFs was counted by light microscopy using 10X magnification.

### Invasion assay

A BioCoat™ Matrigel™ plate with invasion assay inserts (BD Biosciences) was thawed and incubated at RT for 1 h, then 300 μl 0.25% FBS DMEM was applied to the matrigel-coated transwell, and the plate was incubated for 2 h (37 °C, 5% CO_2_). MFs were starved in 0.25% FBS DMEM for 24 h at 37 °C, 5% CO_2_. Cells were trypsinized, centrifuged for 5 min, 1500 x g and suspended in 0.25% FBS DMEM. Cells were counted, and 3 × 10^4^ fibroblasts in 0.25% FBS DMEM were seeded in prepared BD BioCoat™ Matrigel™ transwells, and 0.5 ml of 10% FBS DMEM was added to each well of the plate. The invasion assay was carried out for 24 h at 37 °C, 5% CO_2_. A damp cotton swab was used to wipe the non-invaded cells and matrigel from the top of the transwell membrane. Cells were fixed with 3.7% paraformaldehyde for 15 min, washed twice with PBS, and stained with 0.1% crystal violet. The number of invading MFs was counted by light microscopy using 4X or 10X magnification.

### Luminol hypochlorite assay

Hydrogen peroxide was measured using a GloMax (Promega) microplate reader with injectors, from 200,000 MFs in a 12-well plate in 1 ml of serum-free DMEM utilizing a modified luminol/hypochlorite assay. This assay is based on the oxidation of luminol (5-amino-2,3-dihydro-1,4-phthalazinedione) by sodium hypochlorite (NaOCl) and measures extracellular H_2_O_2_ up to micromolar concentrations [26]. Briefly, DMEM diluted with PBS to 25% was added to a 96-well plate, and luminescence was measured by injecting luminol (Sigma) and sodium hypochlorite (Sigma) to final concentrations of 120 μM and 250 μM, respectively. Hydrogen peroxide concentrations were determined by comparison to experimental standard curves (0 to 100 μM).

## Results

### Murine embryonic fibroblasts (MEFs) and mammary fibroblasts (MFs) deficient in PRDX1 display activated fibroblast and CAF-like characteristics

**Fig. 1:**
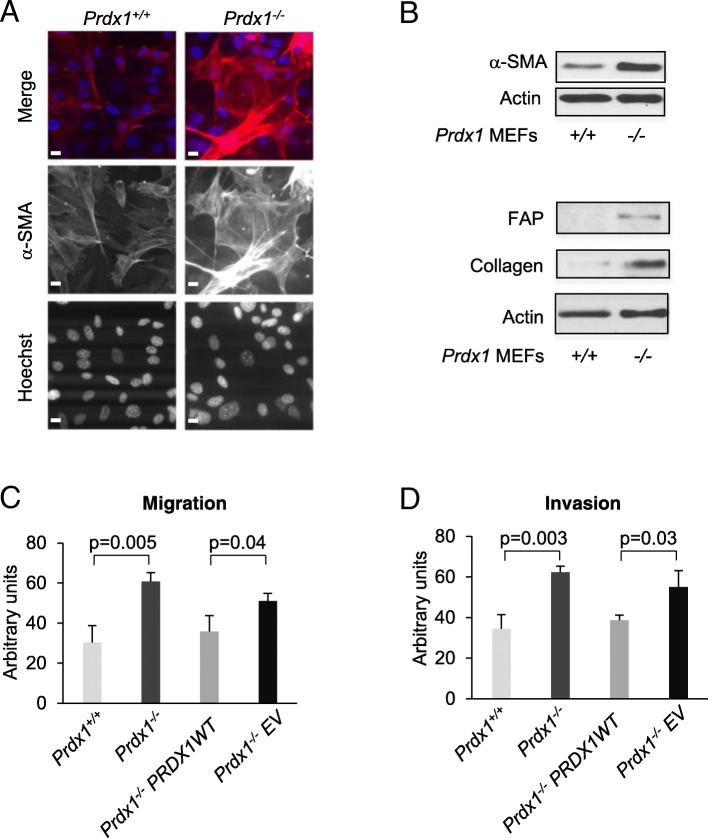
PRDX1-deficient MEFs exhibit characteristics found in myofibroblasts and cancer-associated fibroblasts. **a** MEFs were analyzed for α-SMA (red), Hoechst nuclear stain (blue) using immunofluorescence. Scale bar is 1 μm. **b** MEFs were analyzed for α-SMA or FAP and collagen using immunoblotting. **c** and **d** Transwell migration and invasion assays in *Prdx1*^*−/−*^MEFs reconstituted with wild-type (WT) PRDX1 protein. Values indicate the mean ± SD for 3 repeats. Individual treatment samples were analyzed for statistical significance by Students t-test

To define a potential role for PRDX1 in regulating the activated phenotype in fibroblasts, we investigated MEFs isolated from PRDX1 knockout (*Prdx1*^*−/−*^) and wild-type (*Prdx1*^*+/+*^) embryos [25] for characteristic expression of activated fibroblast and CAF biomarkers. Immunofluorescence (IF) and immunoblotting (IB) experiments showed that loss of PRDX1 increased the levels of α-SMA, procollagen and fibroblast activation protein (FAP) (Fig. 1a and b). As α-SMA and the integral membrane gelatinase FAP participate in migration and invasion during the wound healing processes, we next tested whether the absence of PRDX1 augmented migration and invasion of fibroblasts. *Prdx1*^*−/−*^ MEFs exhibited two-fold higher migration and invasion, which could be rescued by expressing PRDX1 wild-type protein in *Prdx1*^*−/−*^ MEFs (Fig. 1c and d). In all, this suggests that PRDX1 suppresses signaling to promote the activated phenotype in fibroblasts.

**Fig. 2:**
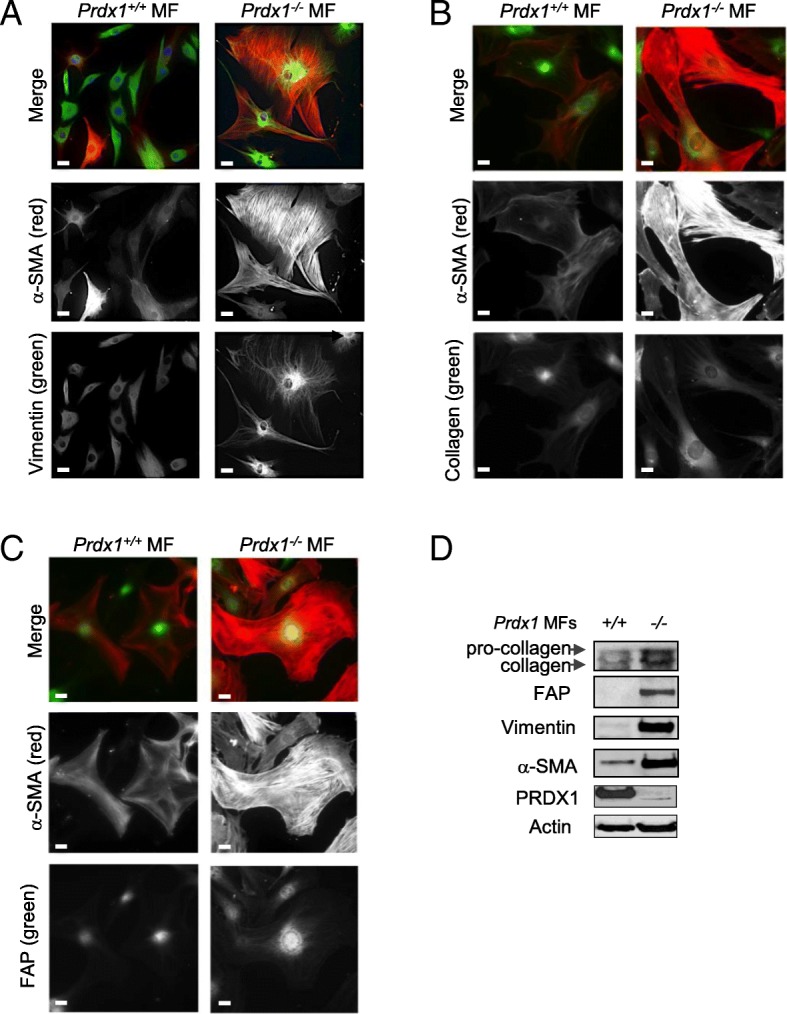
PRDX1-deficiency in MFs induces characteristics found in cancer-associated fibroblasts. **a-c** MFs isolated from female 8-wk-old *Prdx1*^*−/−*^ and *Prdx1*^*+/+*^ mice were analyzed for α-SMA (red), vimentin (green), collagen (green) and FAP (green) by immunofluorescence. Scale bar is 1 μm. **d** MFs were analyzed for α-SMA, vimentin, collagen and FAP proteins by immunoblotting

**Fig. 3:**
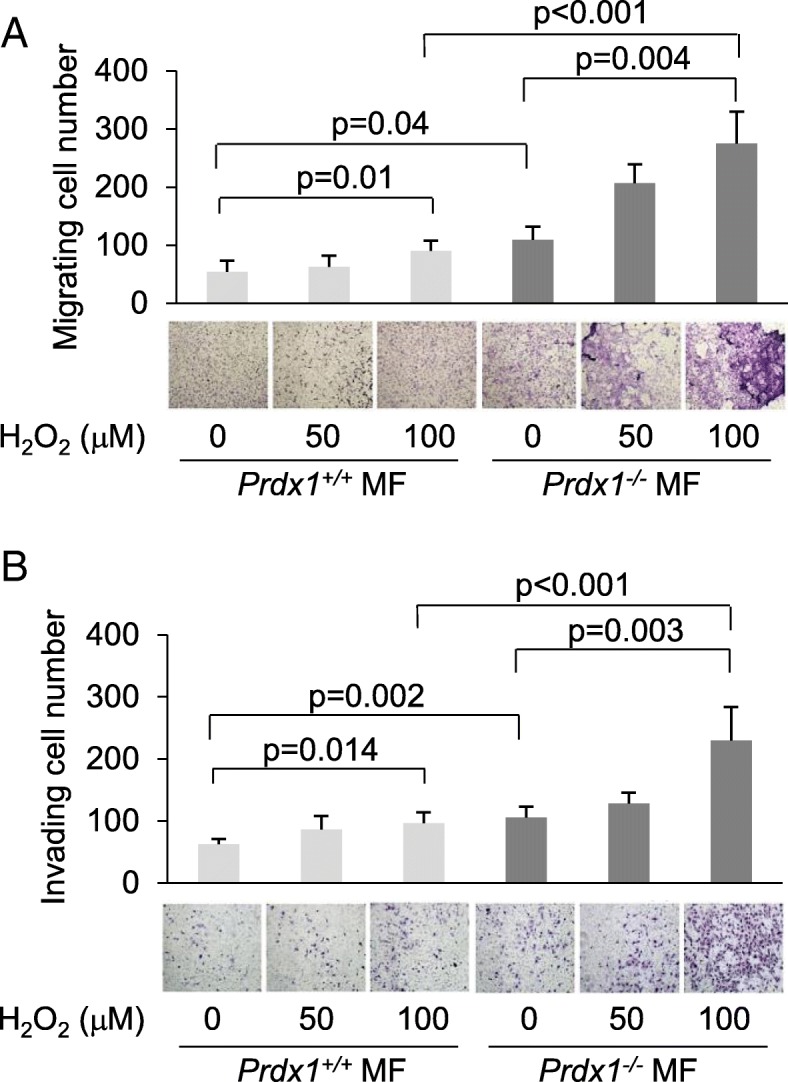
PRDX1-deficient MFs show increased motility and invasion after H_2_O_2_ treatment. **a** and **b.** *Prdx1*^*−/−*^ and *Prdx1*^*+/+*^ MFs were treated with increasing amounts of H_2_O_2_ for 30 min in transwells, H_2_O_2_ was washed off, and transwells were placed into wells. Analysis for migration (**a**) and invasion (**b**) was performed 24 h later

We next examined whether loss of PRDX1 in mammary fibroblasts (MF) led to phenotypic characteristics that resemble cancer-associated fibroblasts (CAFs) [3, 27]. MFs isolated from the mammary glands of 8-wk-old female mice (*Prdx1*^*−/−*^ and *Prdx1*^*+/+*^) were examined by IF and IB. Primary non-immortalized MFs from *Prdx1*^*−/−*^ mice showed increased α-SMA, stress fiber formation, FAP, collagen, and vimentin compared to *Prdx1*^*+/+*^ MFs (Fig. 2a-d and Additional file 1: Figure S1A and B). Activated fibroblasts, as well as CAFs, also display other pro-metastatic behaviors that include enhanced migratory and invasive properties [28]. Utilizing Transwell® migration and invasion assays, *Prdx1*^*−/−*^ MFs exhibited not only higher migration and invasion under basal conditions compared to *Prdx1*^*+/+*^ MFs (1.7-fold and 2-fold, respectively), but H_2_O_2_ treatment increased this difference to 2.4-fold and 3-fold, respectively (Fig. 3a and b).

### PRDX1 deficiency raises H_2_O_2_ levels and activates JNK in MFs

**Fig. 4:**
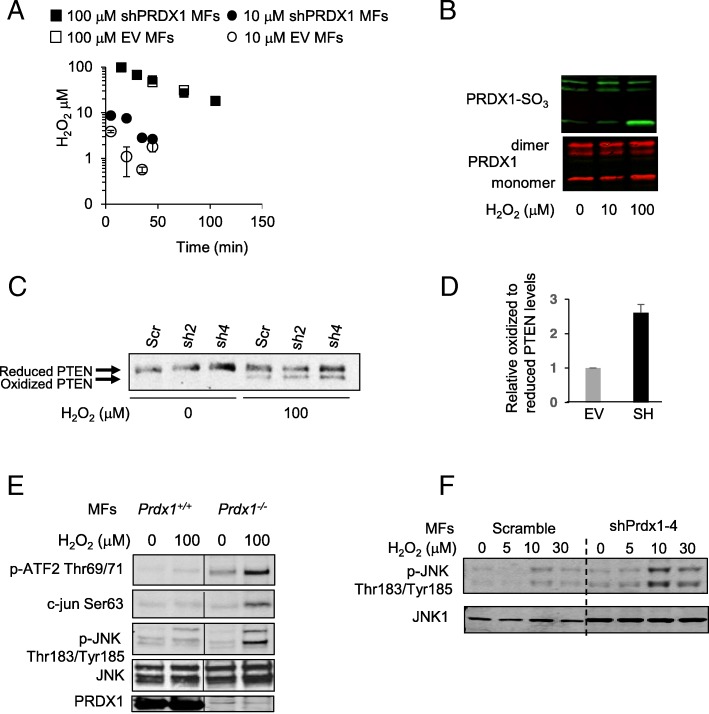
PRDX1 suppresses JNK activation in MFs (**a**) BALB/c MFs were analyzed for H_2_O_2_ scavenging using the luminol hypochlorite assay. (**b**) PRDX1 dimer formation and over-oxidation on Cys52 was analyzed in BALB/c MFs treating cells with H_2_O_2_ as indicated by immunoblotting under non-reducing conditions. (**c** and **d**) Decreased expression of PRDX1 in murine MFs results in PTEN oxidation and intra-disulfide formation in the presence of H_2_O_2_. BALB/c MFs were starved for 24 h in 0.25% FBS DMEM and stimulated with 100 μM H_2_O_2_ for 5 min in the presence of NEM and analyzed under non-reducing conditions by western blotting for PTEN. Scr: scramble, sh2: shPRDx1–2, and sh4: shPRDX1–4 (D) represents the average of relative oxidized to reduced PTEN levels in shPRDx1–2 and shPRDX1–4 expressing cells normalized to EV with STDEV (**e**) *Prdx1*^*−/−*^ and *Prdx1*^*+/+*^ MFs were analyzed by immunoblotting for phosphorylation of c-jun, ATF2 and JNK (**f**) Spontaneously immortalized MFs isolated from 8-wk-old virgin female BALB/c mice were stimulated with 100 μM H_2_O_2_ for 5, 10 and 30 min before analyzed for JNK phosphorylation and JNK1 by immunoblotting

Oxidative stress has been implicated to play an essential role in the activated fibroblast phenotype associated with CAFs [3, 5–8]. To test first whether PRDX1 contributes to H_2_O_2_ homeostasis in MFs, the luminol hypochlorite assay was utilized. MFs were isolated from 8-wk-old female BALB/c mice and infected with retrovirus expressing PRDX1 targeting shRNAs. Two shPRDX1 were tested, shPRDX1–2 and shPRDX1–4, as they reduced PRDX1 expression compared to scramble shRNA 58 and 86%, respectively (Additional file 2: Figure S2A). As expected, shPRDX1–2 and shPRDX1–4 infected MFs exhibited features of the activated phenotype such as increased α-SMA expression and stress fiber formation (Additional file 2: Figure S2B). MFs were treated with low or high dose H_2_O_2_, and peroxide concentrations were measured over time. PRDX1 knockdown MFs decreased H_2_O_2_ levels induced by 100 μM similarly to control MFs, but more slowly when challenged with a lower dose of 10 μM H_2_O_2_ (Fig. 4c). This result implies that PRDX1 is active in MFs and essential for the removal of low dose H_2_O_2_. Accordingly, higher dose H_2_O_2_ was able to over-oxidize PRDX1 in MFs (Fig. 4b).

PTEN is a regulator of CAF activation, and its loss in mammary fibroblasts promotes breast carcinogenesis in mice [21]. Of note, we have recently shown that PRDX1 protects PTEN from H_2_O_2_-induced inactivating oxidation [29]. To test if PTEN intradisulfide formation is increased in MFs lacking PRDX1, shPRDX1–2 and shPRDX1–4 MFs were compared to scramble control cells for increased PTEN intradisulfide formation under non-reducing conditions by SDS-PAGE. As shown in Fig. 4c, both, shPRDX1–2 and shPRDX1–4 MFs displayed higher amounts of PTEN intradisulfides (faster-migrating band) compared to control cells after 100 μM H_2_O_2_ treatment (Fig. 4d). PTEN loss in CAFs activates JNK signaling, and JNK1 stress signaling has been characterized as “hyperactivated” in human fibroblasts isolated from high-density breast tissue samples and the cancer stroma [20, 21]. We, therefore, tested if JNK signaling is affected in MEFs and MFs deficient in PRDX1. Figure 4e and Additional file 2: Figure S2C show PRDX1-deficiency increased phosphorylation of JNK Thr183 and Tyr185 and the transcription factors c-Jun at Ser63 and ATF2 at Thr69 and 71, both of which are specific JNK substrates. Immunoblots of JNK phosphorylation in MFs showed that although knockdown of PRDX1 seemed to result in higher total JNK1 protein levels compared to scramble control (Fig. 4b), JNK phosphorylation was increased at the 30 min time point in the shPRDX1–4 cells compared to scramble control, suggesting that loss of PRDX1 prolongs JNK signaling.

### PRDX1 associates with JNK1 under H_2_O_2_ treatment

**Fig. 5:**
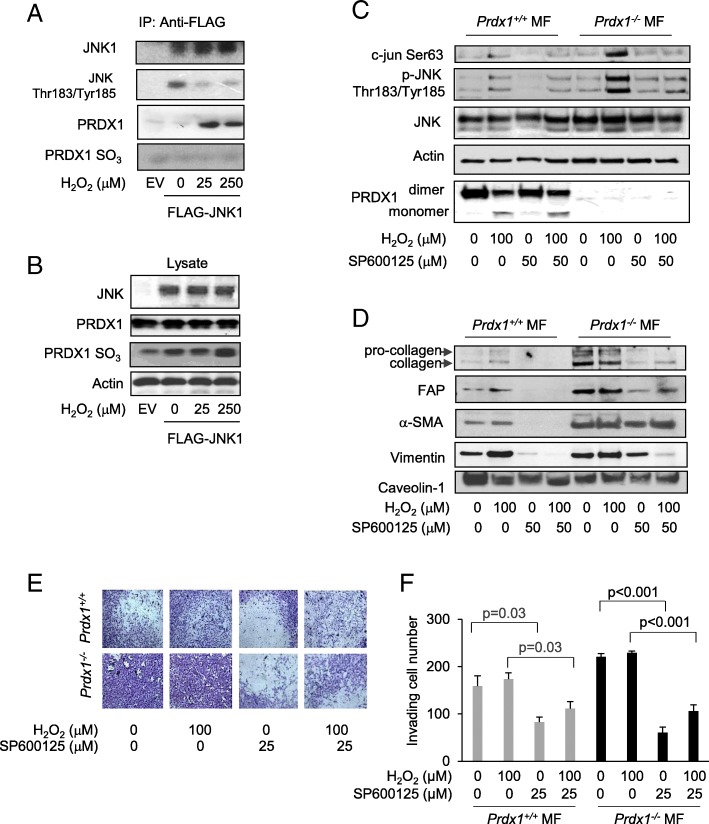
JNK1 associates with PRDX1 under redox (H_2_O_2_) stress and JNK inhibition reduces JNK signaling and characteristics found in CAFs. **a-b** 293 T cells were transfected with pcDNA3-FLAG-JNK1a1 (Addgene) and treated with increasing concentrations of H_2_O_2_ for 30 min. Before lysis, cells were washed with 20 mM N-ethylmaleimide (NEM) in PBS to block lysis-induced disulfide bond formation. FLAG-labeled proteins were immunoprecipitated and detected by immunoblot with FLAG, PRDX1, and PRDXSO2/3 antibodies. Note: JNK1 phosphorylation in the untreated sample may be enhanced by JNK1 over-expression. **b** Protein lysates from (**a**) were analyzed by immunoblotting with antibodies detecting JNK, PRDX1, and PRDXSO2/3. **c** Immortalized *Prdx1*^*−/−*^ and *Prdx1*^*+/+*^ MFs were starved for 48 h before treated with 25 μM SP600125 for 1 h followed by 100 μM H_2_O_2_ treatment for 15 min. Proteins were analyzed by immunoblotting for phosphorylation of c-jun and JNK. **d** Immortalized *Prdx1*^*−/−*^ and *Prdx1*^*+/+*^ MFs were starved for 48 h before treatment with 25 μM SP600125 for 1 h followed by 100 μM H_2_O_2_ treatment for 15 min and analyzed 48 h later for collagen, FAP, α-SMA vimentin, and caveolin 1, which was used as a loading control. **e** and **f** Immortalized *Prdx1*^*−/−*^ and *Prdx1*^*+/+*^ MFs were starved for 48 h before treated with 25 μM SP600125 for 1 h followed by 100 μM H_2_O_2_ treatment for 15 min and analyzed for invasion by Transwell assays. Values indicate the mean ± SD for 3 repeats. Individual treatment samples were analyzed for statistical significance by Students t-test

JNK1 stress signaling is hyperactivated in the cancer stroma [20]. Under ionizing radiation, PRDX1 associates with the GST-pi/JNK1 complex in human lung cancer cells, thereby inhibiting JNK activity [14]. We examined if the PRDX1-JNK1 complex is affected by H_2_O_2_. Immunoprecipitation of FLAG-JNK1 from transfected 293 T cells indicated PRDX1 binding was induced with 25 μM H_2_O_2_ or greater treatment in the presence of lysis buffer containing N-ethylmaleimide (NEM) to reduce lysis induced binding artifacts (Fig. 5a). The binding of PRDX1 to JNK1 coincided with decreased phosphorylation of JNK1 on Thr183 and Tyr185, supporting the finding from Kim et al. where PRDX1 binding suppresses JNK activity [14]. Additionally, overoxidation of the catalytic Cys52 in PRDX1 observed following treatment with heightened H_2_O_2_ in cell lysates was not detected in PRDX1-JNK1 complexes (Fig. 5b), suggesting that JNK activity might increase with over-oxidized PRDX1 under H_2_O_2_ stress.

### Inhibition of JNK signaling reduces CAF-like characteristics

**Fig. 6:**
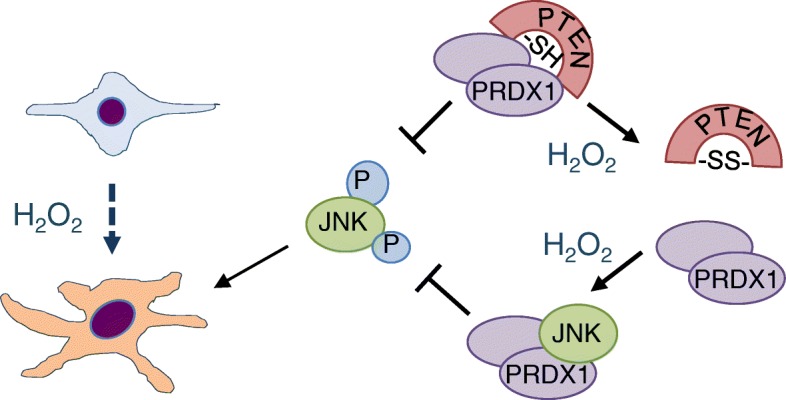
PRDX1 acts as a gatekeeper of JNK activity in the transition of benign fibroblasts into CAFs under rising levels of H_2_O_2_. PRDX1 controls JNK activation under low H_2_O_2_ levels through protecting PTEN from inactivation by H_2_O_2_. As H_2_O_2_ levels rise, PRDX1 dissociates from PTEN resulting in reversible PTEN inactivation [29, 30] and JNK phosphorylation and activation [31]. Under these circumstances, PRDX1 binds associates with JNK and inhibits JNK activation. Therefore, it is proposed that PRDX1 controls JNK activity on two levels: first, by promoting PTEN activity and secondly, by binding directly to JNK

We next examined if JNK inhibition can reduce the CAF-like phenotype in *Prdx1*^−/−^ MFs. SP600125, a reversible ATP competitive JNK inhibitor, inhibited H_2_O_2_-induced phosphorylation of c-jun and JNK in *Prdx1*^*−/−*^MFs to levels found in *Prdx1*^*+/+*^MFs (Fig. 5c). Treatment of MFs with 100 μM H_2_O_2_ for 15 min increased expression of collagen, FAP, α-SMA and vimentin protein levels in 48 h in *Prdx1*^*+/+*^ MFs, but not in *Prdx1*^*−/−*^ MFs (Fig. 5d). SP600125 treatment repressed this increased expression (Fig. 5d). As collagen, vimentin, α-SMA and FAP are involved in cell migration and invasion, the effect of JNK inhibition on invasion was assessed. *Prdx1*^*+/+*^ MFs and *Prdx1*^*−/−*^ MFs were pretreated with 25 μM of SP600125 before exposure to 100 μM of H_2_O_2_ and analyzed by Transwell® invasion assay. JNK inhibition decreased invasion of non-peroxide treated *Prdx1*^*+/+*^ MFs by 48% and by 36% in H_2_O_2_-treated *Prdx1*^*−/−*^ MFs (Fig. 5e). JNK inhibition similarly decreased invasion of non-peroxide treated *Prdx1*^*−/−*^ MFs by 73 and 54% in H_2_O_2_-treated *Prdx1*^*−/−*^ MFs (Fig. 5e and f). In conclusion, the data shown here suggest that PRDX1 controls the transition of benign fibroblasts into CAFs not only by promoting PTEN activity, thus decreasing JNK downstream signaling, but also by directly inhibiting JNK1 itself (Fig. 6).

## Discussion

We show for the first time an involvement of the peroxidase PRDX1 in regulating the activated phenotype of mammary fibroblasts that resembles the transition from normal fibroblasts into CAFs. The involvement of oxidative stress in the form of H_2_O_2_ has long been implicated in the transition of normal fibroblasts into CAFs, thereby creating an oxidative stress field effect within the tumor [3, 5, 7, 32]. Inhibition of NOX4-derived H_2_O_2_ has been suggested as a method to block the activated phenotype in lung fibroblasts to reverse the molecular pathogenesis of fibrosis [33]. Oxidative stress is pathogenic in mammary fibroblasts as it promotes the transition from normal to cancer-associated fibroblast. Based on the present findings, we propose a model where oxidative stress-induced transition of normal MFs in to CAFs is protected by PRDX1. Under normal H_2_O_2_ homeostasis, JNK activation is prevented by PRDX1 binding to PTEN to protect it from oxidation-induced activation [29]. Once H_2_O_2_ levels rise, PRDX1 dissociates from PTEN which then can lead to JNK activation. However, since PRDX1 associates with JNK under these circumstances, JNK activation and thus the transition of normal MFs into CAFs is inhibited (Fig. 6).

c-Jun N-terminal kinase activation is intimately involved in regulating myofibroblast activation, and its role in MFs has started to emerge. For example, deletion of PTEN in MFs caused activation of AKT and JNK signaling, which is upstream of the ETS2 transcription factor [21, 34]. PTEN is a redox reactive lipid and tyrosine phosphatase that is sensitive to reversible inactivation by oxidation of cysteine 124 in its active site [30, 35]. As mutation, truncation or other genomic variations are rare in CAFs compared to epithelial cells, it is very likely that loss of PTEN function in CAFs stems from posttranslational modification such as oxidation of its catalytic cysteine, as we and others have shown [29, 30, 36]. High dose H_2_O_2_ results in dissociation of PRDX1 from PTEN and PTEN inactivation, which might occur more frequently in the microenvironment of breast cancer compared to the normal breast, since ROS are known to be higher in cancer tissues [37]. This suggests that under higher or constant oxidative stress, PTEN can be inactivated by oxidation in MFs thereby contributing to breast carcinogenesis. We recently demonstrated that PRDX1 regulates MAPK signaling by selectively modulating MKP1 and MKP5 phosphatases, which regulate JNK and p38 MAP kinases, respectively [24]. Like PTEN, MKP1 and MKP5, belong to the protein tyrosine phosphatase family, which transfer phosphates through a nucleophilic cysteine in their active sites [38]. PRDX1 protects MKP1 and MKP5 from oxidation-induced inactivation in an H_2_O_2_-dose-dependent manner. While PRDX1 protects MKP1 only under low H_2_O_2_ levels, PRDX1 supports MKP-5 activation under low and high doses of H_2_O_2_ [24, 29, 30]. As PRDX1-deficient MFs show increased JNK activation, this may reflect low MKP-1 activity, due to oxidation-induced inactivation. Future studies are required to determine if PRDX1 inactivation contributes to JNK signaling in CAFs through loss of MKP1 protection.

During cardiac overload-induced hypertrophy, JNK1 mediates α-SMA expression that is essential in cardiac remodeling and vascular smooth muscle activity [39–41]. In the heart, the infarct border zone is infiltrated with abundant stress fiber localized α-SMA-positive myofibroblasts resulting in scar contraction and synthesis of large amounts of extracellular matrix [42]. In the skin, α-SMA expression in fibroblasts induces contractility and tissue stiffness [43]. While JNK induced α-SMA is beneficial in wound healing, it has an opposite effect in breast cancer, where tissue stiffness, partially caused by CAF activities, leads to invasion and metastasis. JNK may, therefore, emerge as a therapeutic target in CAFs as it has in lung fibrosis [19]. In support of our findings shown here, JNK modulates H_2_O_2_ levels by regulating the NADPH oxidase 4 (NOX4) thereby promoting the activated phenotype in MFs [18]. Additional evidence for JNK’s active role in mammary stroma comes from a study interrogating the genome-wide transcriptional profiling of low and high-density human breast tissues. Comparison of specific gene set enrichment analysis from this study to breast tumor stroma revealed that JNK1 stress signaling is the single most significant biological process that is shared between these two data sets [20]. Taken together, this strongly suggests that JNK1 is a driver of stroma remodeling and supports our finding of reduced α-SMA, FAP and vimentin expression in the presence of the JNK inhibitor SP600125.

Similar to CAFs, activated fibroblasts that cause fibrosis exhibit different properties from myofibroblasts that promote wound healing, as the latter die after the wound has closed [44]. Therefore, fibrotic fibroblast and CAF-like phenotypes may be closely linked in some cancers. For example, 80% of all hepatocellular carcinomas originate from fibrotic livers [45]. Similarly, myofibroblasts found in wound healing and fibrosis, activated fibroblasts as well as CAFs express vimentin, α-SMA, and FAP, secrete collagen, form contractile stress fibers, remodel the ECM through proteolytic enzymes and their inhibitors and are motile as well as invasive [43–46]. However, differences in response to apoptotic stimuli, autophagy, and redox signaling have been suggested to account for the differenence between myofibroblasts and activated fibroblasts or tumor-associated fibroblasts [3]. Cancer is a wound that never heals. In that sense, future studies are needed to define the role of ROS in wound healing versus the CAF activated phenotype. Our data suggest that JNK signaling is involved in regulating the invasive nature of CAFs. Although studies suggest that α-SMA and collagen are direct targets of JNK signaling, the dipeptidyl peptidase and collagenase FAP has yet to be realized as activated by JNK signaling, although the promoter contains two AP-1 binding sites (http://genome.ucsc.edu) [19, 40, 41]. In fibroblasts as well as endothelial cells, chemical and genetic ablation of JNK controls a wide range of targets in metastasis signaling, including the expression of metalloproteinases, motility and invasion [47–57].

## Conclusion

In summary, we demonstrate for the first time that JNK is regulated by PRDX1 in the transition of activated fibroblasts into CAFs and therefore may be a new potential drug target in stroma rich cancers such as breast cancer. Also, our data suggest that inhibiting PRDX1 as cancer therapeutic may inactivate PTEN and activate JNK thereby promoting the CAF phenotype.

## Additional files

Additional file 1: **Figure S1:** PRDX1-deficiency in MFs induces characteristics found in cancer-associated fibroblasts. MFs isolated from female 8-wk-old *Prdx1*^*−/−*^ and *Prdx1*^*+/+*^ mice were analyzed for α-SMA (red), vimentin (red) and Hoechst nuclear stain (blue) by IF. Scale bar in 1 μm. (PPTX 2482 kb)

Additional file 2: **Figure S2:** Knockdown of PRDX1 in MFs results in characteristics found in CAFs and JNK activation. **(A)** Spontaneously immortalized MFs isolated from 8-wk-old virgin female Balb/c mice were infected with lentiviruses expressing 4 different shPRDX1 targeting shRNAs and analysis by immunoblotting for PRDX1 expression. **(B)** α-SMA (red) staining and light microscopy of Balb/c MFs expressing EV or shPRDX1. Scale bar in 1 μm. **(C)** *Prdx1*^*−/−*^ and *Prdx1*^*+/+*^ MEFs were analyzed by immunoblotting for phosphorylation of c-jun, ATF2 and JNK. (PPTX 520 kb)

## Data Availability

The datasets used and/or analyzed during the current study available from the corresponding author on reasonable request.

## Abbrevations

JNK: Jun-c terminal kinase
Prdx1: Peroxiredoxin1
MEFs: Murine embryonic fibroblasts
CAF: Cancer-associated fibroblast
MF: Mammary fibroblast
MMPs: Matrix metalloproteinases
FAP: Fibroblast activation protein
α-SMA: alpha smooth muscle actin
ROS: Reactive oxygen species
H_2_O_2_: Hydrogen peroxide
