# Supplementary material for: The peroxidase PRDX1 inhibits the activated phenotype in mammary fibroblasts through regulating c-Jun N-terminal kinases
Source: BMC Cancer. 2019 Aug 16;19:812. doi: 10.1186/s12885-019-6031-4 (PMC6697950; doi:10.1186/s12885-019-6031-4)
Supplement: Supplementary file 1 — Figure S1: PRDX1-deficiency in MFs induces characteristics found in cancer-associated fibroblasts. MFs isolated from female 8-wk-old Prdx1−/− and Prdx1+/+ mice were analyzed for α-SMA (red), vimentin (red) and Hoechst nuclear stain (blue) by IF. Scale bar in 1 μm. (PPTX 2482 kb) [file 12885_2019_6031_MOESM1_ESM.pptx]

## Slide 1
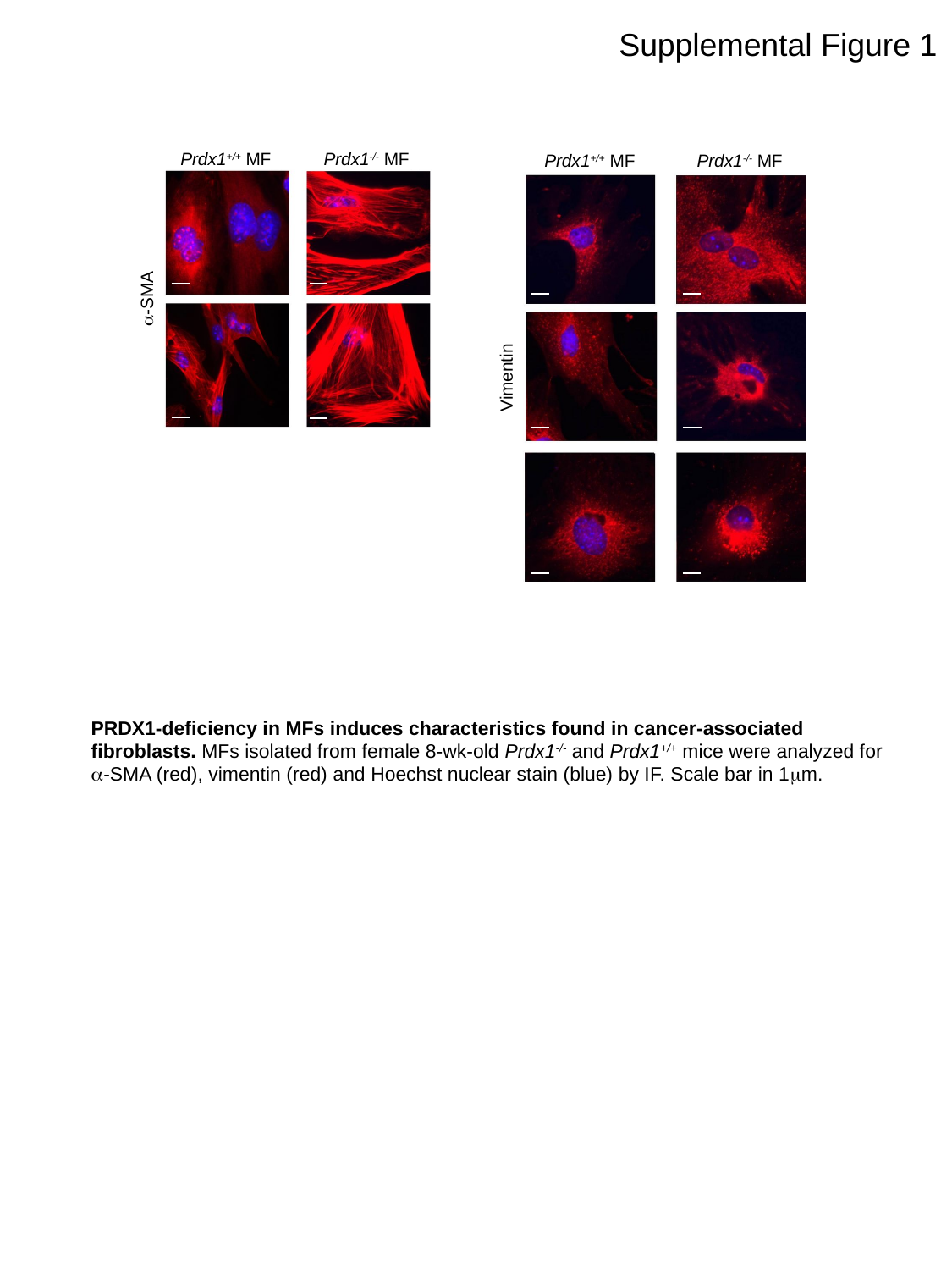

Supplemental Figure 1
Prdx1+/+ MF
Prdx1-/- MF
Prdx1+/+ MF
Prdx1-/- MF
a-SMA
Vimentin
PRDX1-deficiency in MFs induces characteristics found in cancer-associated fibroblasts. MFs isolated from female 8-wk-old Prdx1-/- and Prdx1+/+ mice were analyzed for a-SMA (red), vimentin (red) and Hoechst nuclear stain (blue) by IF. Scale bar in 1mm.
