# Supplementary material for: The peroxidase PRDX1 inhibits the activated phenotype in mammary fibroblasts through regulating c-Jun N-terminal kinases
Source: BMC Cancer. 2019 Aug 16;19:812. doi: 10.1186/s12885-019-6031-4 (PMC6697950; doi:10.1186/s12885-019-6031-4)
Supplement: Supplementary file 2 — Figure S2: Knockdown of PRDX1 in MFs results in characteristics found in CAFs and JNK activation. (A) Spontaneously immortalized MFs isolated from 8-wk-old virgin female Balb/c mice were infected with lentiviruses expressing 4 different shPRDX1 targeting shRNAs and analysis by immunoblotting for PRDX1 expression. (B) α-SMA (red) staining and light microscopy of Balb/c MFs expressing EV or shPRDX1. Scale bar in 1 μm. (C) Prdx1−/− and Prdx1+/+ MEFs were analyzed by immunoblotting for phosphorylation of c-jun, ATF2 and JNK. (PPTX 520 kb) [file 12885_2019_6031_MOESM2_ESM.pptx]

## Slide 1
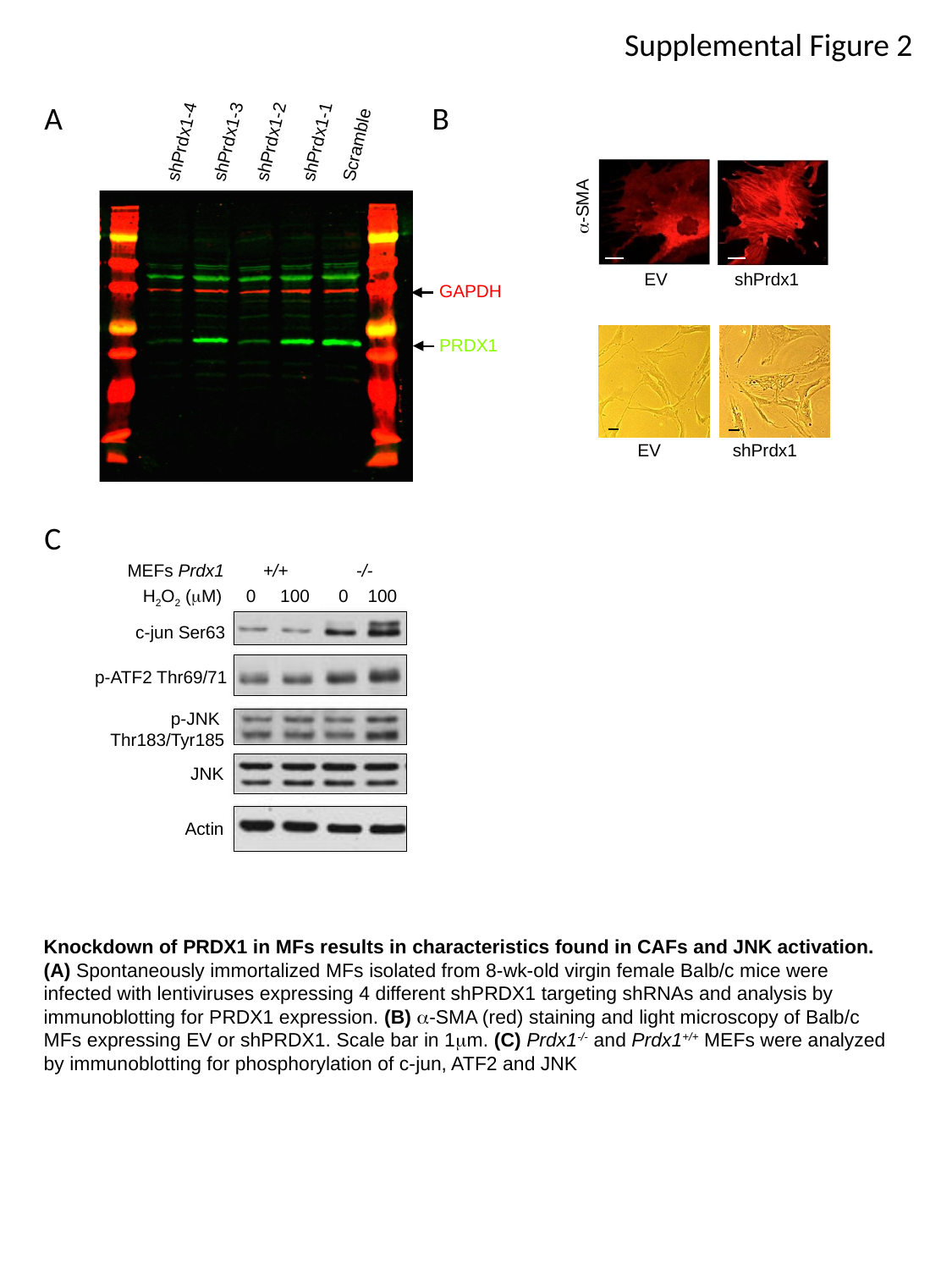

Supplemental Figure 2
shPrdx1-4
shPrdx1-3
shPrdx1-2
shPrdx1-1
Scramble
GAPDH
PRDX1
A
B
a-SMA
EV
shPrdx1
EV
shPrdx1
C
MEFs Prdx1 +/+ -/-
JNK
Actin
H2O2 (mM) 0 100 0 100
c-jun Ser63
p-ATF2 Thr69/71
p-JNK
Thr183/Tyr185
Knockdown of PRDX1 in MFs results in characteristics found in CAFs and JNK activation. (A) Spontaneously immortalized MFs isolated from 8-wk-old virgin female Balb/c mice were infected with lentiviruses expressing 4 different shPRDX1 targeting shRNAs and analysis by immunoblotting for PRDX1 expression. (B) a-SMA (red) staining and light microscopy of Balb/c MFs expressing EV or shPRDX1. Scale bar in 1mm. (C) Prdx1-/- and Prdx1+/+ MEFs were analyzed by immunoblotting for phosphorylation of c-jun, ATF2 and JNK
